# Impact of exogenous hydrogen peroxide on osteogenic differentiation of broiler chicken compact bones derived mesenchymal stem cells

**DOI:** 10.3389/fphys.2023.1124355

**Published:** 2023-01-26

**Authors:** Y. H. Tompkins, G. Liu, W. K. Kim

**Affiliations:** Department of Poultry Science, University of GA, Athens, GA, United States

**Keywords:** bone health, oxidative stress, chicken MSCs, cellular ROS, cell differentiation

## Abstract

The effects of hydrogen peroxide (H_2_O_2_) on the osteogenic differentiation of primary chicken mesenchymal stem cells (MSCs) were investigated. MSCs were subjected to an osteogenic program and exposed to various concentrations of H_2_O_2_ for 14 days. Results showed that high concentrations of H_2_O_2_ (200 and 400 nM) significantly increased pro-apoptotic marker *CASP8* expression and impaired osteogenic differentiation, as indicated by decreased mRNA expression levels of osteogenesis-related genes and reduced *in vitro* mineralization. In contrast, long-term H_2_O_2_ exposure promoted basal expression of adipogenic markers at the expense of osteogenesis in MSCs during osteogenic differentiation, and increased intracellular reactive oxygen species (ROS) production, as well as altered antioxidant enzyme gene expression. These findings suggest that long-term H_2_O_2_-induced ROS production impairs osteogenic differentiation in chicken MSCs under an osteogenic program.

## Introduction

Modern commercial poultry production is strictly operated based on balanced nutrition and optimized environmental conditions. However, oxidative stress is ubiquitous in broiler production systems. For example, unbalanced nutrition, pathogen infection, and poor environmental conditions including heat stress, ammonia exposure, and flock density, can induce oxidative stress in broilers (Mishra and Jha, 2019; Ali Hassan and Li, 2021; Chauhan et al., 2021). Oxidative stress has been reported as a negative factor in broiler performance, healthy growth, and production quality (Mishra and Jha, 2019; Surai et al., 2019), representing an unbalanced condition between the production of reactive oxygen species (ROS) and the antioxidant defense systems (Sies et al., 2017). In broiler production, in addition to management-associated physiological oxidative stress, infectious agents are key factors that can cause severe oxidative stress in broilers (Zorov et al., 2014; Forrester et al., 2018).

Bone growth depends on the activity of bone-related cells, where osteoblasts are important cells involved in bone formation (Hambli, 2014). Osteoblast originates from mesenchymal stem cells (Zomorodian and Baghaban Eslaminejad, 2012). There is an ongoing interest in using mesenchymal stem cells (MSCs) as a study model to understand cell physiology, and etiology of bone disease (Svoradova et al., 2021), and the effect of oxidative stress on mammal MSCs has been well noted (Denu and Hematti, 2016). Hydrogen peroxide (H_2_O_2_), a non-radical ROS, has been extensively studied for its effects (Sharma et al., 2012). It is one of the most common endogenous byproducts of mitochondrial respiration that is present in the avian system (Ojano-Dirain et al., 2007). At a cellular level, ROS level is tightly regulated by the antioxidant defense system and is critical for MSCs multipotency (Atashi et al., 2015). A low basal level of ROS is a critical mediator in pathophysiological responses (Maraldi et al., 2015), while differentiated MSCs presented a higher intracellular ROS production which is essential for cell survival and early differentiation (Hu et al., 2018). However, the uncontrollable high levels of ROS not only impair the cell membrane fluidity and permeability but are also responsible for oxidation damage to DNA, RNA, protein, and lipid damage in mitochondria, leading to cellular senescence including cell dysfunction, cellular injury and cell apoptosis (Denu and Hematti, 2016). For cellular homeostasis, endogenous scavengers such as enzymatic proteins, including superoxide dismutase (SOD), glutathione peroxidase (GPx), and catalases (CAT), and non-enzymatic antioxidants, such as vitamins and trace minerals (Hu et al., 2018), are all work together for controlling intracellular ROS-related stress by removing and converting excessive ROS (Denu and Hematti, 2016).

Relatively high level of ROS can directly interact with critical signaling molecules in essential osteogenic pathways, negatively impacts bone homeostasis (Atashi et al., 2015). Oxidative stress induced by ROS over-production has been considered a pathogenic factor involved in human skeletal disorders (Sharma et al., 2015). Moreover, in response to oxidative stress, ROS can activate extracellular signal-regulated kinases that modulate nuclear factor kappa light chain enhancer of activated B-cell (NF-κB) and NF-E2 p45-related factor 2 (NRF2) signaling pathways, which are critical for regulating inflammation, cellular redox status, and bone homeostasis by regulating osteoblast or osteoclast differentiation and activity (Sun et al., 2015; Yuan et al., 2017). For example, in the mouse cell line, ROS induced MSCs commit to adipogenesis rather than osteogenesis at the transcriptional level (Lin et al., 2018). However, the response of chicken MSCs to high ROS levels and the effect of ROS production on avian osteoblastic differentiation are not well understood. Understanding the impact of ROS on MSC terminal fate and differentiation capacity is important for developing novel strategies for prevent, anticipate, or revert oxidative stress-induced leg problems in chicken production. Therefore, in the current study, H_2_O_2_ was used as a stimulator of oxidative stress in MSCs culture. This study aimed to investigate the effects of H_2_O_2_ on the osteogenic differentiation of chicken MSCs isolated from broiler compact bones.

## Materials and methods

### Animal use and ethics statement

The study was carried out in compliance with the ARRIVE guidelines. All experiment protocols and animal use were approved by the Institutional Animal Care and Use Committee at the University of Georgia, Athens, GA.

### Isolation of broiler MSCs

MSCs were isolated using previously described methods (Adhikari et al., 2018). Briefly, legs from one-day old chicks were obtained after cervical dislocation. The leg tissue was soaked in alcohol for a minute and then dried with Kimwipes (Kimberly Clark, Irving, TX, United States). Muscle was removed, and long bones were harvested. The long bones were kept in high glucose Dulbecco’s Modified Eagle’s medium (DMEM; contains 4.5 g/L glucose, 25 mM HEPES, sodium pyruvate, and without L-glutamine; 15–018-cv, Corning, Corning, NY, United States) until muscle and cartilage tissues were completely removed using a scalpel and scissors in a bio-safety cabinet (NuAire, Plymouth, MN, United States). The bones were placed in washing buffer to cut off the metaphysis. Only tibia diaphysis and femur diaphysis were kept for cell isolation. Washing buffer contained 2% fetal bovine serum (FBS) (Hyclone Laboratories Inc., Logan, UT) in Dulbecco’s phosphate-buffer saline (PBS) (Corning). The bones were cracked with a scalpel, bone marrow was flushed out with washing buffer, and bone marrow was discarded. The bones were chopped into small fragments and suspended in a 50 mL tube containing a 10 mL digestion medium consisting of 100 IU/mL penicillin, 100 ug/mL streptomycin, 0.25% collagenase (Sigma-Aldrich, St. Louis, MO, USA), 20% FBS, and high glucose DMEM. The tubes were placed in a 37°C incubated with an orbital shaker set at 180 rpm for 60 min (VWR, Radnor, PA, United States). The digested bone solution was filtered with a 40 μm cell strainer (Thermo Fisher Scientific, Waltham, MA, United States) set over a 50 ml tube to remove the bone fragments, and then the filtered medium was centrifuged at 1,200 rpm for 10 min. The supernatant was discarded and the cell pellet was resuspended in 20 ml growth medium consisting of DMEM with 10% FBS, 100 U/mL penicillin, 100 μg/mL streptomycin, and 0.292 mg/mL L-glutamine (Thermo Fisher Scientific), and 10 ml resuspend cells were plated in a 100 mm cell culture dishes (Corning). Cells were incubated at 37°C in a humidified incubator (NuAire) containing 5% CO_2_. Half of the medium was replaced with fresh growth medium after 24 h of culture, and the culture medium was changed every two days thereafter. For cell passing, when the cells reached 80% confluency, they were washed twice with 5 mL pre-warmed PBS, dissociated with 1.5 mL 0.1% Trypsin-EDTA (Corning) for 2 min at 37°C, and subcultured with cell density of 25,000 cells/cm^2^ in 100 mm cell culture dishes.

### Viability of cultured chicken MSCs with H_2_O_2_ exposure

The viability of cells was determined using cellular 3-(4,5-dimethylthiazol-2-yl)-2,5-diphenyltetrazolium bromide (MTT) kits (Cayman Chemical, Ann Arbor, MI, USA). Cells were seeded at a concentration of 5 × 10^4^ cells/100 μL in differentiation medium per well with 96-well black wall culture plates. The treatment of MSCs with various concentrations of H_2_O_2_ (50, 100, 200, 400, and 800 nM; H_2_O_2_, 30% (w/w) solution, Sigma-Aldrich) during culture was also examined. The H_2_O_2_ stock was diluted by PBS, and the same volume of diluted H_2_O_2_ solution was added to the culture medium. MSCs were cultured without H_2_O_2_ but PBS (osteogenic differentiation medium with 0 nM H_2_O_2_) as a control. Cells were incubated with different levels of H_2_O_2_ treatments for 6, 24, and 48 h in the dark. The MTT viability assay was not performed for longer periods of time because high cell density led to high absorbance readings that impaired the accuracy of detection. DMEM with 10% MTT was added and incubated for 4 h, after wich the culture medium was completely discarded. The formed formazan was dissolved with 100 µl dimethyl sulfoxide (DMSO; Sigma-Aldrich) to produce a purple color, and the plates were then placed on an orbital shaker (VWR) set at low speed for 5 min. The absorbance was measured at 570 nm using a microplate reader (BioTek, Winooski, VT, United states).

### Intracellular reactive oxygen species detection

The intracellular ROS levels were examined using the DCFDA/H_2_DCFDA cellular ROS assay kits (Abcam Cambridge, MA, United states)) according to the manufacturer’s instruction. Briefly, as per manufacturer’s instructions, 3 × 10^4^ cells were seeded in a black clear-flat-bottom 96-well microplate and allowed to adhere overnight. The cells were treated with or without H_2_O_2_ treatment for 2 h, 6 h, 24 h, and 48 h, but not for longer periods of time as the ROS detection required relatively low cell density for accurate results. After the corresponding treatments, the medium was removed, 100 µL/well of 1× washing buffer was added to remove any residual material, and then 100 µL/well of the diluted DCFDA solution was added to stain for 45 min at 37°C in the culture incubator. After removing the DCFDA solution, the cells were rinsed once with a washing buffer, 100 µL/well of washing buffer was then added for microplate measurement. The fluorescence density was measured with a microplate reader (Spectramax M5, Molecular Devices, San Jose, CA) at an excitation wavelength of 485 nm and an emission wavelength of 535 nm. Images were obtained using a fluorescence microscope (Keyence bz-X8000, Keyence Corp., Osaka, Japan) at ×10 magnification.

### Osteogenic differentiation

MSC cells were expanded to passage 4 for osteogenic differentiation study. After selecting the proper concentration for H_2_O_2_ treatment based on the MTT assay, the cells were seeded at a density of 8 × 10^4^ cells per well in 0.2% gelatin-coated (Alfa Aesar, Ward Hill, MA USA) 24-well cell culture plates (Corning), and cultured in growth medium at 37°C in the cell culture incubator (NuAire) until 100% confluency. The cells were then treated with osteogenic differentiation medium containing high glucose DMEM with 10^–7^ M dexamethasone (Sigma-Aldrich), 10 mM β-glycerophosphate (Sigma-Aldrich), 50 μg/mL ascorbate (Sigma-Aldrich), 5% FBS, and 100 U/mL penicillin, 100 μg/mL streptomycin, and 0.292 mg/mL L-glutamine (Thermo Fisher Scientific) to induce osteogenic differentiation. Cells cultured in growth medium served as the negative control. Culture medium was replaced with fresh pre-warmed differentiation medium daily. The cells underwent differentiation for 6 h, 24 h, 48 h, 72 h, 96 h, 5 days, 6 days, 10 days and 14 days.

### Alizarin red S staining and mineral deposit quantification

The degree of mineralization of chicken MSCs was determined using Alizarin red S staining (Adhikari et al., 2018). Briefly, the cells were seeded at a density of 8 × 10^4^ cells per well in 0.2% gelatin-coated (Alfa Aesar) 24-well cell culture plates (Corning), and cultured in a growth medium at 37°C in the cell culture incubator (NuAire) until 100% confluency. The cells were then exposed to H_2_O_2_ in osteogenic differentiation medium for 6, 10, and 14 days. On each day of staining, the cells were fixed with 10% neutral buffered formalin for 1 h and then stained with 0.2% Alizarin red S (Sigma-Aldrich, St. Louis, MO) in distilled water for 45 min at room temperature. After rinsing with distilled water, images of cell culture plates were captured in ×2 magnification using a microscope with a camera (Keyence bz-×8000, Keyence). Mineralized nodules were labeled as dark red spots. To quantify the mineral deposition, the stained cells were solubilized with 200 µl of 10% acetic acid per well and incubated for 30 min with low-speed shaking on an orbital shaker (VWR). After the monolayer was loosely attached, the cells were gently scraped from the plate and transferred to a 1.5 mL microcentrifuge tube. The microcentrifuge tubes containing the cells were then vortexed vigorously for 40 s and heated to 85 °C for 10 min. The tubes were transferred on ice to cool down for 5 min and then centrifuged at 20,000 g for 15 min. After that, 150 µl of the supernatant was aliquoted to a new 1.5 ml microcentrifuge tube and the pH was neutralized with 60 µl 10% ammonium hydroxide. After supernatant neutralization, 50 µl of each sample was loaded into an opaque-walled transparent bottom 96-well plate and read at OD 405 nm using a microplate reader (BioTek) for Alizarin red S staining quantification (Serguienko et al., 2018).

### Von kossa staining and quantification

The degree of mineralization of chicken MSCs was determined using the von Kossa staining (Adhikari et al., 2018). Cells were seeded at a density of 8 × 10^4^ cells per well in 0.2% gelatin-coated (Alfa Aesar) 24-well cell culture plates (Corning), and cultured in growth medium at 37°C until 100% confluency. Then the cells were exposed to H_2_O_2_ in osteogenic differentiation medium for 6, 10, and 14 days. At different time points, the cell culture plates were washed three times with PBS and then fixed with 0.1% glutaraldehyde (G5882, Sigma-Aldrich) in PBS (pH 7.0) for 15 min at room temperature. After discarding the fixation buffer, the cells were washed three times with distilled water and then incubated in 5% silver nitrate (Sigma-Aldrich) for 30 min. The silver nitrate solution was discarded, and the cells were washed with distilled water at least three times, air-dried, and exposed to bright light until black color developed in areas of calcification. Images of cell culture plates were captured at ×2 magnification using a microscope with a camera (Keyence bz-×8000, Keyence). Mineralized nodules were observed as dark brown to black spots. The stained plates were quantified using the area fractions method with the ImageJ program (National Institutes of Health, Bethesda, MD, USA). Three images from each well were analyzed, and the mean area fraction from each well was used for statistical analysis.

### RNA isolation, cDNA synthesis, and real-time polymerase chain reaction (qRT-PCR) analysis

The cell culture process for RNA isolation was the same as the osteogenic differentiation. Briefly, MSC cells were expanded to passage 4 and seeded at a density of 8 × 10^4^ cells per well in 0.2% gelatin-coated (Alfa Aesar) 24-well cell culture plates (Corning), and cultured in growth medium until they reached100% confluency. The cells were differentiated for 6 h, 24 h, 48 h, 72 h, 96 h, 5 days, 6 days, 10 days and 14 days, with or without H_2_O_2_ treatment. At each time point, total RNA was extracted from the cells using QIAzol lysis reagents (Qiagen 79,306, Germantown, MD, USA) according to the manufacturer’s instructions. The Nano-Drop 1000 Spectrophotometer (ThermoFisher Scientific) was used to determine the quantity of extracted RNA. cDNA was synthesized from 2000 ng of total RNA using high-capacity cDNA reverse transcription kits (Thermo Fisher Scientific). Quantitative real-time reverse transcription polymerase chain reaction (qRT-PCR) was used to measure mRNA expression. Primers were designed using the Primer-BLAST program (https://www.ncbi.nlm.nih.gov/tools/primer-blast/). The specificity of primers was validated by PCR product sequencing; the details of primer sequences used for the experiment are presented in Table 1. Primer quality was verified through melting curve analysis and gel electrophoresis in this study. The qRT-PCR was performed on an Applied Biosystems StepOnePlus™ (Thermo Fisher Scientific) with iTaq™ universal SYBR Green Supermix (BioRad, Hercules, CA, United states) using the following conditions for all genes: 95°C for 10 min followed 40 cycles at 95°C for 15 s, annealing temperature (Table 1) for 20 s, and extending at 72°C for 1 min.

**TABLE 1:** Nucleotide sequences of the primers used for quantitative real-time RT-PCR.

Gene1	Primer sequence (5′-3′	Product length (bp)	Annealing temperature (°C)	Accession #
18S rRNA	F-AGCCTGCGGCTTAATTTGAC	121	56.5	AF_173612.1
	R-CAACTAAGAACGGCCATGCA			
HMBS	F-GGCTGGGAGAATCGCATAGG	131	59	XM_004947916.3
	R-TCCTGCAGGGCAGATACCAT			
ACTB	F-CAACACAGTGCTGTCTGGTGGTA	205	61	NM_205518.1
	R-ATCGTACTCCTGCTTGCTGATCC			
C/EBPa	F-CCTACGGCTACAGAGAGGCT	206	60	NM_001031459.1
	R-GAAATCGAAATCCCCGGCCA			
PPARG	F-GAGCCCAAGTTTGAGTTTGC	131	58	XM_025154400.1
	R-TCTTCAATGGGCTTCACATTT			
FABP4	F-GCAGAAGTGGGATGGCAAAG	153	60	NM_204290.1
	R-GTTCGCCTTCGGATCAGTCC			
ALPL	F-CGACCACTCACACGTCTTCA	140	60	NM_205360.1
	R-CGATCTTATAGCCAGGGCCG			
RUNX2	F-ACTTTGACAATAACTGTCCT	192	60	XM_015285081.2
	R-GACCCCTACTCTCATACTGG			
BGLAP	F-GGATGCTCGCAGTGCTAAAG	142	57	NM_205387.3
	R-CTCACACACCTCTCGTTGGG			
SPP1	F-GCCCAACATCAGAGCGTAGA	204	57	NM_204535.4
	R-ACGGGTGACCTCGTTGTTTT			
BMP2	F-TCAGCTCAGGCCGTTGTTAG	163	57	XM_025148488.1
	R-GTCATTCCACCCCACGTCAT			
COL1A2	F- CTG​GTG​AAA​GCG​GTG​CTG​TT	222	60	NM_001079714.2
	R-CACCAGTGTCACCTCTCAGAC			
SOD2	F- GCC​ACC​TAC​GTG​AAC​AAC​CT	140	61	NM_204211.2
	R- AGT​CAC​GTT​TGA​TGG​CTT​CC			
SOD1	F-ATTACCGGCTTGTCTGATGG	173	58	NM_205064.1
	R-CCTCCCTTTGCAGTCACATT			
CAT	F-ACTGCAAGGCGAAAGTGTTT	222	60	NM_001031215.1
	R-GGCTATGGATGAAGGATGGA			
GSTa	F- GAG​TCA​ATT​CGG​TGG​CTG​TT	157	59	XM_046913335.1
	R- TGC​TCT​GCA​CCA​TCT​TCA​TC			
NOS2	F-CCTGTACTGAAGGTGGCTATTGG	66	58	NM_204961.2
	R-AGGCCTGTGAGAGTGTGCAA			
GPX1	F-AACCAATTCGGGCACCAG	122	60	NM_001277853.2
	R-CCGTTCACCTCGCACTTCTC			
NFR2	F- GAG​CCC​ATG​GCC​TTT​CCT​AT	210	59	XM_046907885.1
	R- CAC​AGA​GGC​CCT​GAC​TCA​AA			
CASP3	F-TGGTATTGAAGCAGACAGTGGA	103	60	XM_015276122.2
	R-GGAGTAGTAGCCTGGAGCAGTAGA			
CASP8	F-ATTTGGCTGGCATCATCTGT	146	59	NM_204592.4
	R-ACTGCTTCCCTGGCTTTTG			
CASP6	F-AAACCTACACCAACCACCACA	196	60	NM_001396146.1
	R-TTCTGTCTGCCAAAGTCCCA			

^a^18S rRNA:18 S ribosomal RNA; HMBS: hydroxymethylbilane synthase; ACTB: actin beta; PPARG: peroxisome proliferator-activated receptor gamma; C/EBPα: CCAAT/enhancer-binding protein alpha; FABP4: fatty acid binding protein 4; SPP1: secreted phosphoprotein, osteopontin; BMP2: bone morphogenetic protein two; BGLAP: bone gamma-carboxyglutamic acid-containing protein (osteocalcin); RUNX2: runt-related transcription factor 2; ALPL: alkaline phosphatase, biomineralization associated; COL1A2: collagen type I alpha two chain; CAT: catalase; SOD1: superoxide dismutase one; SOD2: superoxide dismutase two; GPX1: glutathione peroxidase one; NOS2: nitric oxide synthase two; NFR2: GA, binding protein transcription factor alpha subunit (GABP2); GSTa: GSTA2, glutathione S-transferase alpha two; CASP3: caspase three; CASP6: caspase six; CASP8: caspase 8.

The geometric means of the cycle threshold (Ct) values of three housekeeping genes, including hydroxymethylbilane synthase (*HMBS*), 18 S ribosomal RNA (*18S rRNA*), and actin beta (*ACTB*) were used for normalization. The stability of the housekeeping genes was confirmed by their consistent Ct values among the treatments (*p* > 0.1) and also assessed by statistical algorithms by software program NormFinder (Version 0.953; https://moma.dk/normfinder-software) (Wan et al., 2011). Peroxisome proliferator-activated receptor gamma (*PPARG*), adipose tissue fatty acid binding protein 4 (*FABP4*), and CCAAT enhancer binding protein alpha (*CEBPA*) were used as early markers of adipogenic differentiation, and alkaline phosphatase-biomineralization associated (*ALPL*), bone gamma-carboxyglutamate protein (*BGLAP*), runt-related transcription factor 2 (*RUNX2*), secreted phosphoprotein 1 (*SPP1*), collagen type I alpha two chain (*COL1A2*), bone–specific alkaline phosphatase (*ALP*), and bone morphogenetic protein 2 (*BMP2*) were used as osteogenic marker genes in the bone marrow. Nuclear factor kappa B subunit 1 (*NFKB1*) and antioxidant enzyme protein coding genes including catalase (*CAT*), superoxide dismutase type 1 (*SOD1*), superoxide dismutase type 2 (*SOD2*), glutathione peroxidase 1 (*GPX1*), glutathione S-transferase alpha 2 (*GSTA2*), and nitric oxide synthase 2 (*NOS2*) were used to determine the antioxidant enzyme activity and oxidative stress status. Pro-apoptotic marker genes, such as Caspase 3 (*CASP3*), Caspase 8 (*CASP8*), and Caspase 6 (*CASP6*), were used to assess the cell apoptosis. Samples were run in triplicate, and relative gene expression data were analyzed using the 2^−ΔΔCt^ formula (Livak and Schmittgen, 2001). The expression levels of the other treatment groups were presented as fold changes relative to the average ΔCT value for each gene in the control group.

### Statistical analysis

All experimental data were expressed as means with standard errors of the mean (SEM). The data were tested for homogeneity of variances and normality of studentized residuals. The differences between the treatment groups were analyzed using one-way ANOVA, and the means were statistically analyzed using Tukey’s test using JMP Pro14 (SAS Institute, Cary, NC, USA). Statistical significance was set at *p ≤* 0.05, and values of 0.05 ≤ *p* ≤ 0.1 were also presented to show a trend towards statistical significance (Serdar et al., 2021).

## Results

### Cell viability, cell apoptosis, and intracellular ROS production with exogenous H_2_O_2_ exposure

**FIGURE 1:**
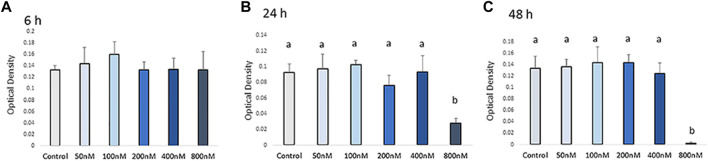
Effects of H_2_O_2_ on the cell viability. Cells were treated with the indicated concentrations of H_2_O_2_ for 6 h **(A)**, 24 h **(B)** and 48 h **(C)**. The graphs show changes in cellular growth as assessed by MTT assays. The MTT assay showed that exposure to concentrations higher than 400 nM of H_2_O_2_ can reduce cell viability. Therefore, the appropriate H_2_O_2_ concentration was screened out and final treatment concentrations of 100 nM, 200 nM and 400 nM of H_2_O_2_ were selected as the treatments dose for the following experiments. ^a, b^ Treatments with different letters indicate a significantly difference between treatments using Tukey’s HSD test, *p* < 0.05; data are shown as mean ± SEM of four independent replicates (*n* = 4).

Cell viability was measured by MTT assay after H_2_O_2_ exposure (Figure 1). The viability of chicken MSCs exposed to H2O2 showed a different rate of decline among doses. Chicken MSCs were cultured with different concentrations (50–800 nM) of H_2_O_2_ in osteogenic differentiation medium for 6 h showed a non-cytotoxic effect (*p* > 0.05; Figure 1A). At 24 h of treatment, 800 nM of H_2_O_2_ significantly reduced cell viability (*p* < 0.05; Figure 1B) by approximately 70% compared to the untreated control. After 48 h, 800 nM of H_2_O_2_ reduced cell viability by approximately 90% compared to the untreated control (*p* < 0.05; Figure 1C). No statistically significant change in cell viability was observed with treatment concentrations lower than 800 nM. Treatment doses above 400 nM significantly reduced the number of cells to an extent that was not adequate to conduct the experiment. Therefore, doses below 400 nM were selected for further studies. The final treatment concentrations of 100, 200, and 400 nM of H_2_O_2_ were selected as the treatment doses for the following experiments.

**FIGURE 2:**
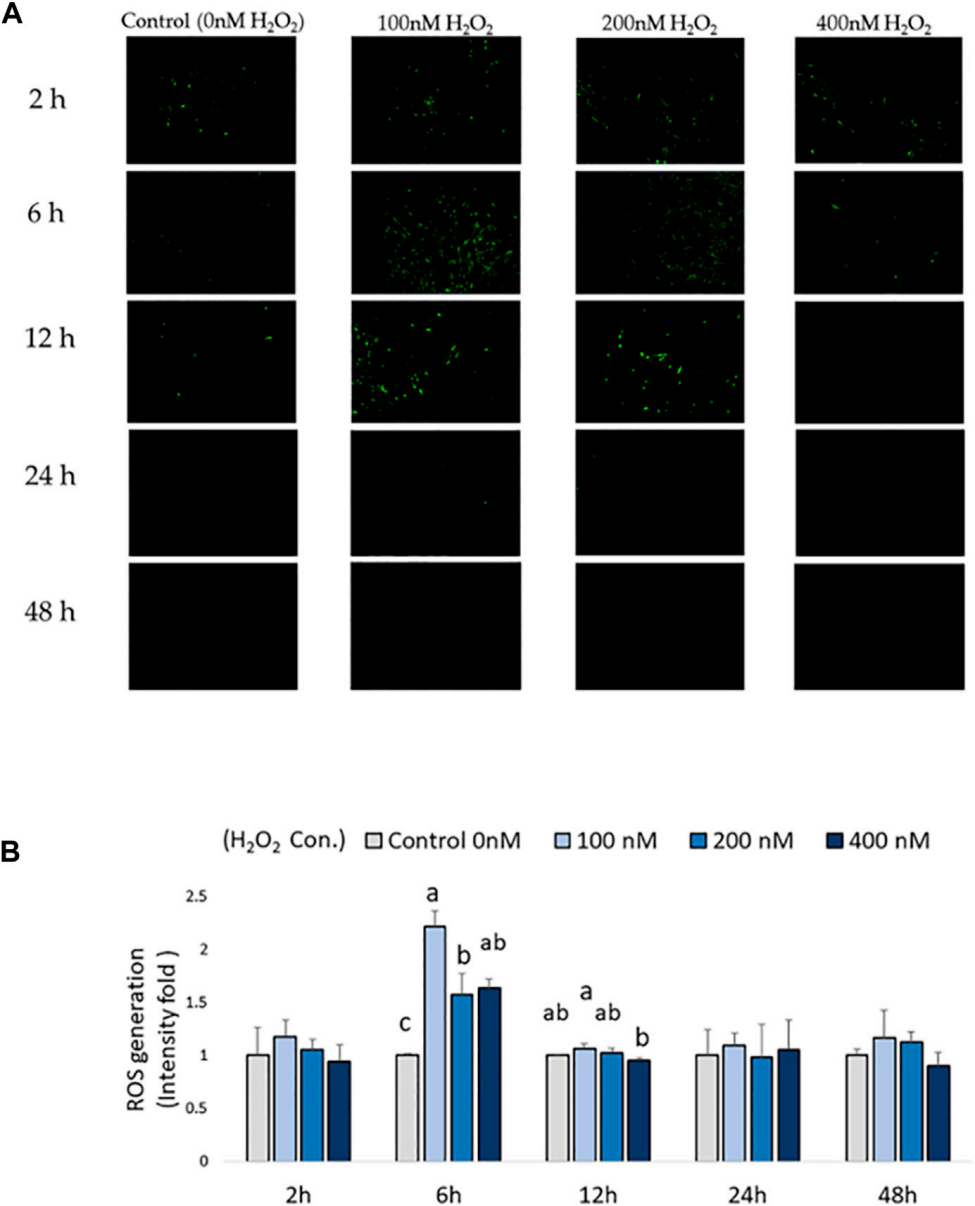
Effects of H_2_O_2_-induced reactive oxygen species (ROS) production in chicken MSCs. 3 × 10^4^ cells were seeded in a black clear-flat-bottom 96-well microplate and allowed to adhere overnight. Cells were then treated with the indicated concentrations of H_2_O_2_ for 2 h, 6 h, 12 h, 24 h and 48 h. ROS levels in MSCs were measured using a DCFDA/H_2_DCFDA cellular ROS assay kit. MSCs cultured without H_2_O_2_ but PBS (osteogenic differentiation medium with 0 nM H_2_O_2_) were used as the control. **(A)** Figures were selected as representative images from the DCFDA/H_2_DCFDA cellular ROS assay at different time points. MSCs cultured without any treatments or DCFDA/H_2_DCFDA was used to set the background adjustment. **(B)** Quantitative analysis was performed by measuring fluorescence intensity. Each value represents the mean ± SEM of three independent replicates (*n* = 3). MSCs cultured in osteogenic differentiation medium without H_2_O_2_ treatment (0 nM H_2_O_2_) were used as the control, and data were present as fold-change normalized to the fluorescence intensity level of the control. ^a, ab, b, c^ Treatments with different letters indicate a significantly difference between treatments using Tukey’s HSD test, *p* < 0.05.

Intracellular ROS production in chicken MSCs after H_2_O_2_ exposure was monitored using a cellular DCFDA assay (Figures 2A,B). A significant increase in intracellular ROS production was detected in 100, 200, and 400 nM H_2_O_2_-treated chicken MSCs after 6 h compared to the control (*p* < 0.05; Figure 2B), with the highest upregulated ROS response observed at 100 nM of H_2_O_2_. After 12 h of H_2_O_2_ exposure, there was a significant difference in ROS production between 100 nM and 400 nM groups (*p* < 0.05; Figure 2B). However, intracellular ROS signal were not significantly affected by H_2_O_2_ treatment after 24 and 48 h of H_2_O_2_ exposure. Based on these data, the effective treatment duration is 12 h following exogenous H_2_O_2_ exposure. To minimized damage to the cell membrane and reduce variations in results due to cell death and reduced cell number caused by prolonged severe stress, the frequency of medium change was set to be every 24 h.

**FIGURE 3:**
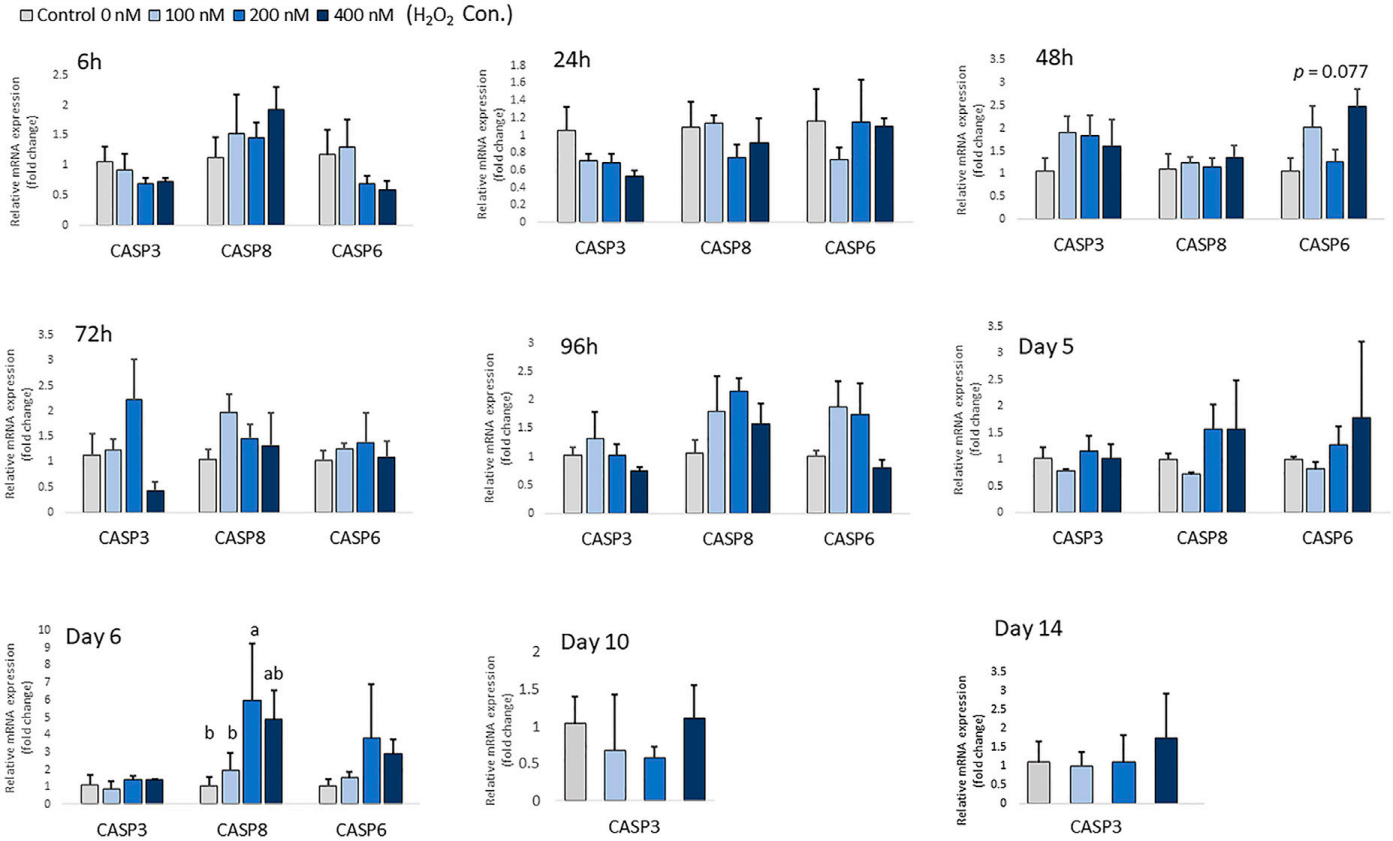
Effects of H_2_O_2_ on mRNA expression of apoptosis markers in chicken MSCs. Differentiation cells were treated with the indicated concentrations of H_2_O_2_ for 6 h, 24 h, 48 h, 72 h, 96 h, 5 days, 6 days, 10 days and 14 days. Each value represents the mean ± SEM of three independent experiments (*n* = 3). CASP3: caspase three; CASP6: caspase six; CASP8: caspase eight; ^a, ab, b, c^ Treatments with different letters indicate a significantly difference between treatments using Tukey’s HSD test, *p* < 0.05.

At low concentrations of H_2_O_2_ treatment, mRNA expression of pro-apoptotic markers, including *CASP-3*, *CASP-6* and *CASP-8* remained unchanged in chicken MSCs during the early differentiation stage from 6 h to day 5 (*p* > 0.05; Figure 3). This suggests that the effect of H_2_O_2_ on the osteogenic differentiation of MSCs was not due to a cytotoxic effect causing cell apoptosis at the early differentiation stage. However, 200 nM H_2_O_2_ significantly increased mRNA expression of *CASP-8* compared to the control on day 6 (*p* < 0.05; Figure 3), and 400 nM H_2_O_2_ numerically increased expression of *CASP-8* (*p* < 0.05; Figure 3). Neither of the higher treatment doses changed the expression of *CASP-3* or *CASP-*6 on day 6. On day 10 and day 14, high cycle threshold (Ct > 38) value indicating the expression of *CASP-6* and *CASP-8* were not detected.

### Altered gene expression of antioxidant enzyme in response to extracellular H_2_O_2_ exposure

**FIGURE 4:**
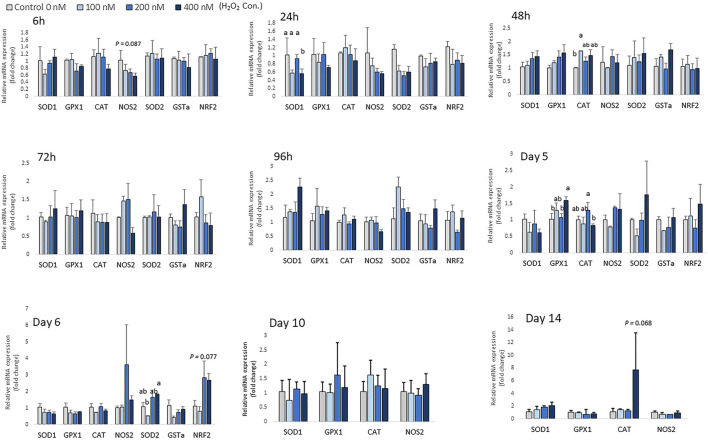
Effects of H_2_O_2_ on mRNA expression of antioxidant enzymes in chicken MSCs. Cells were treated with the indicated concentrations of H_2_O_2_ for 6 h, 24 h, 48 h, 72 h, 96 h, 5 days, 6 days, 10 days and 14 days. The expression of *SOD2*, *GSTa* and *NRF2* was not detected on day 10 and day 14 of differentiation. Each value represents the mean ± SEM of three independent experiments (*n* = 3). CAT: catalase; SOD1: superoxide dismutase one; SOD2: superoxide dismutase two; GPX1: glutathione peroxidase one; NOS2: nitric oxide synthase two; NFR2: GA binding protein transcription factor alpha subunit (GABP2); GSTa: glutathione S-transferase alpha two; ^a, ab, b^ Treatments with different letters indicate a significantly difference between treatments using Tukey’s HSD test, *p* < 0.05.

There was no significant change in the expression of antioxidant enzyme mRNA after 6 h of differentiation, except for expression of *NOS2*, which showed a trend of decreasing with higher H_2_O_2_ treatment doses (*p =* 0.087; Figure 4). At 24 h of treatment, the mRNA expression of *SOD1* was decreased by the highest concentration of H_2_O_2_ (400 nM) (*p* < 0.05; Figure 4). At 48 h of treatment, 100 nM H_2_O_2_ augmented the expression of *CAT* compared to the control. After 5 days of treatment and differentiation, the mRNA expression of *GPX1* was upregulated by 400 nM H_2_O_2_ compared to the control (*p* < 0.05). 200 nM H_2_O_2_ significantly increased mRNA expression of *CAT* compared to the 400 nM H_2_O_2_ treatment group (*p* < 0.05). After 6 days of H_2_O_2_ treatment, 400 nM H_2_O_2_ significantly upregulated mRNA expression of *SOD2* compared to the 100 nM H_2_O_2_ group (*p* < 0.05). There was a trend of increasing expression of *NRF2* with 200 and 400 nM H_2_O_2_ treatments (*p* = 0.077). After 14 days of differentiation, a trend of increasing mRNA level of *CAT* (*p* = 0.068) was observed with 400 nM H_2_O_2_ treatment. However, expression of *SOD2*, *GSTa* and *NRF2* was not detected on day 10 and day 14 of differentiation. In conclusion, depending on the different treatment concentration of H_2_O_2_, the expression of antioxidant enzyme gene was altered at the later stage of differentiation.

### Effect of H_2_O_2_ on osteogenesis in chicken MSCs

**FIGURE 5:**
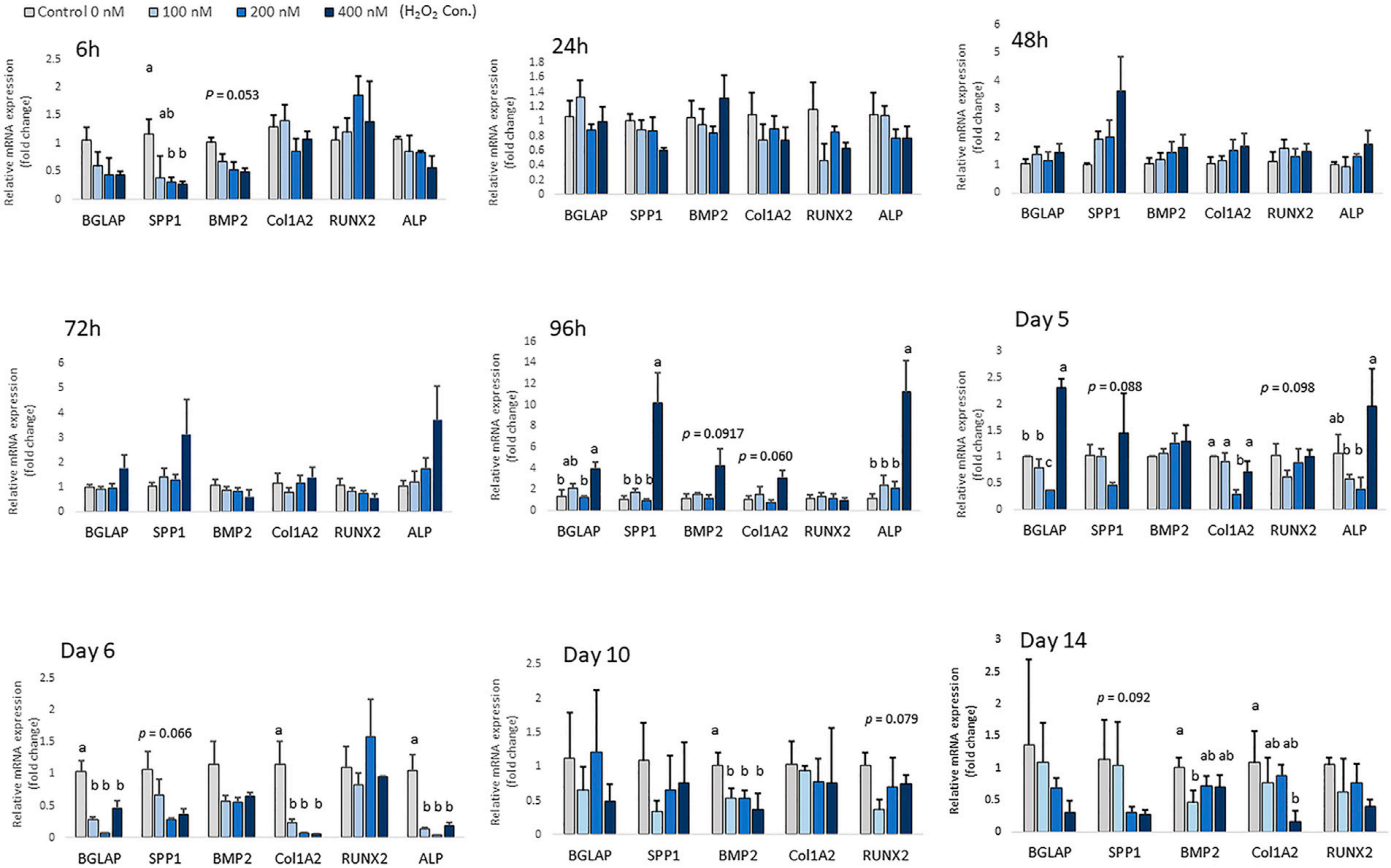
Effects of H_2_O_2_ on mRNA expression of osteogenic differentiation markers in chicken MSCs. Cells were treated with the indicated concentrations of H_2_O_2_ in differentiation medium for 6 h, 24 h, 48 h, 72 h, 96 h, 5 days, 6 days, 10 days and 14 days. Each value represents the mean ± SEM of three independent experiments (n = 3). SPP1: secreted phosphoprotein, osteopontin; BMP2: bone morphogenetic protein two; BGLAP: bone gamma-carboxyglutamic acid-containing protein (osteocalcin); RUNX2: runt-related transcription factor 2; ALP: alkaline phosphatase, biomineralization associated; Col1A2: collagen type I alpha two chain. ^a, ab, b, c^ Treatments with different letters indicate a significantly difference between treatments using Tukey’s HSD test, *p* < 0.05.

Chicken MSCs were treated with various concentrations of H_2_O_2_ in osteogenic differentiation medium for 14 days, the effects on osteogenic differentiation varied at different time points (Figure 5). At the beginning of differentiation, there was a significant decrease in *SPP1* (*p* < 0.05) and a numerically decreased mRNA expression of *BMP2 (p* = 0.053) after 6 h of H_2_O_2_ treatment_._ In the following days, the mRNA expression of osteogenic marker genes was unchanged. After 96 h of treatment, cells exposure to 400 nM H_2_O_2_ showed significantly higher mRNA expression of *ALP* (*p* < 0.05), *BGLAP* (*p* < 0.05) and *SPP1* (*p* < 0.05), and 400 nM H_2_O_2_ tended to increase the expression of *BMP2* (*p* = 0.092) and *Col1A2* (*p* = 0.060). After 5 days of H_2_O_2_ exposure, mRNA expression of *BGLAP* (*p* < 0.05), *ALP* (*p* < 0.05) and *Col1A2* (*p* < 0.05) was suppressed by 200 nM H_2_O_2_ compared to the control. In contrast, mRNA expression of *BGLAP* was upregulated by 400 nM H_2_O_2_ compared to the other treatment groups (*p* < 0.05) and the expression of *ALP* was increased by 400 nM H_2_O_2_ (*p* < 0.05) compared to the low treatment doses of 100 and 200 nM H_2_O_2_. After 6 days of daily treatment, mRNA expression of *ALP* (*p* < 0.05), *BGLAP* (*p* < 0.05) and *Col1A2* (*p* < 0.05) was reduced by H_2_O_2_ treatments, and there was a trend of decreasing in *SPP1* (*p* = 0.066) with H_2_O_2_ treatments. On day 10, the expression of *BMP2* was significantly reduced by H_2_O_2_ treatments compared to the control (*p* < 0.05), and there was a trend of decreasing in *RUNX2* (*p* = 0.079) with H_2_O_2_ treatments. On day 14, 100 nM H_2_O_2_ significantly reduced the expression of *BMP2* compared to the control (*p* < 0.05), and 400 nM H_2_O_2_ significantly reduced expression of *Col1A2* compared to the control (*p* < 0.05). There was also a trend of decreasing in *SPP1* (*p* = 0.092) with H_2_O_2_ treatments on day 14. However, *ALP* expression was not detected on day 10 and day 14 of differentiation.

**FIGURE 6:**
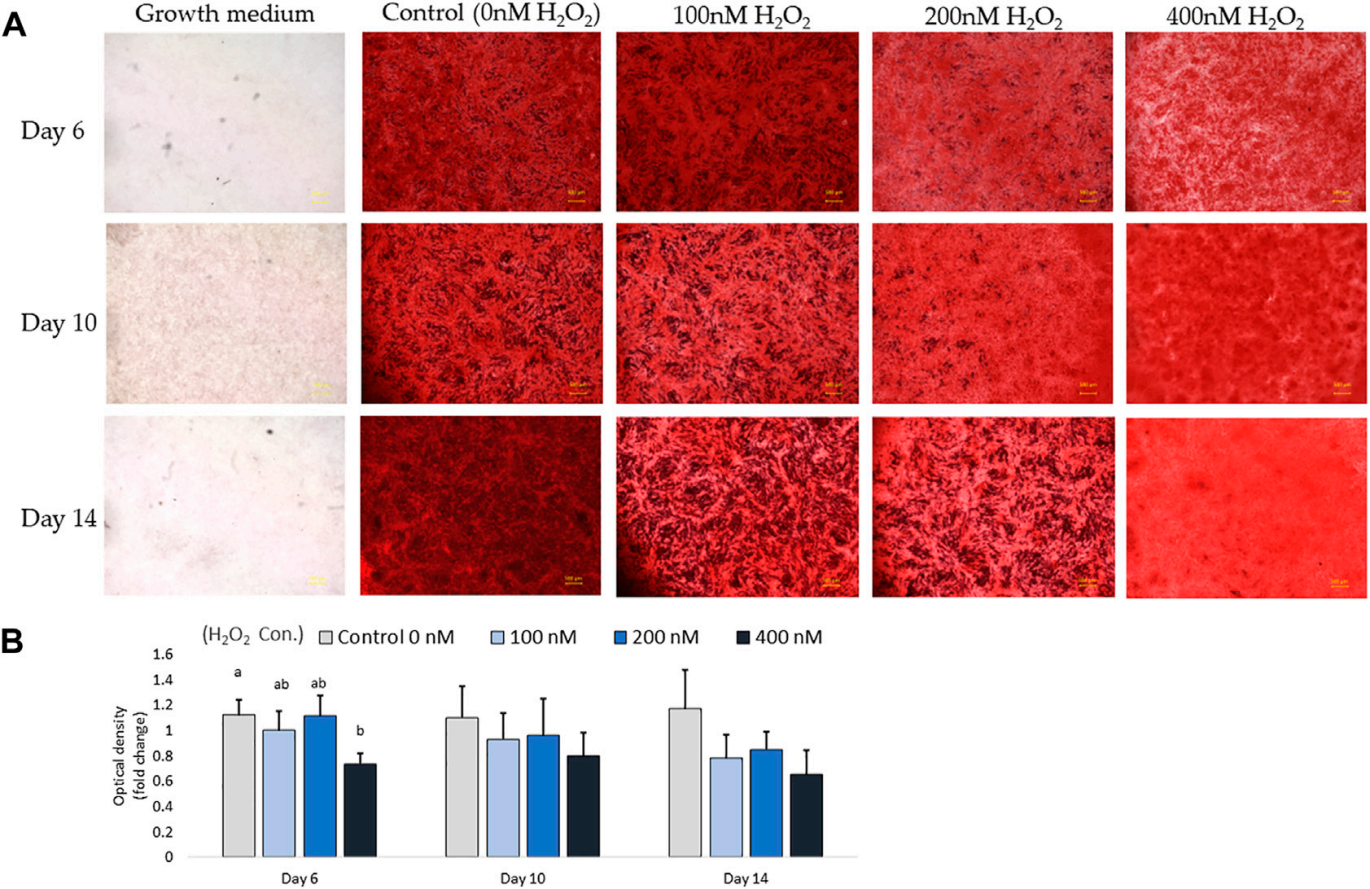
Alizarin red S staining for mineralization on day 6, day 10 and day 14. Images were randomly acquired at ×2 magnification. The calcified nodules appeared bright red in color. Mineral deposit quantification was conducted, with each value representing the mean ± SEM of three independent experiments (*n* = 3). ^a, b^ Treatments with different letters indicate a significantly difference between treatments using Tukey’s HSD test at each time points, respectively, *p* < 0.05.

**FIGURE 7:**
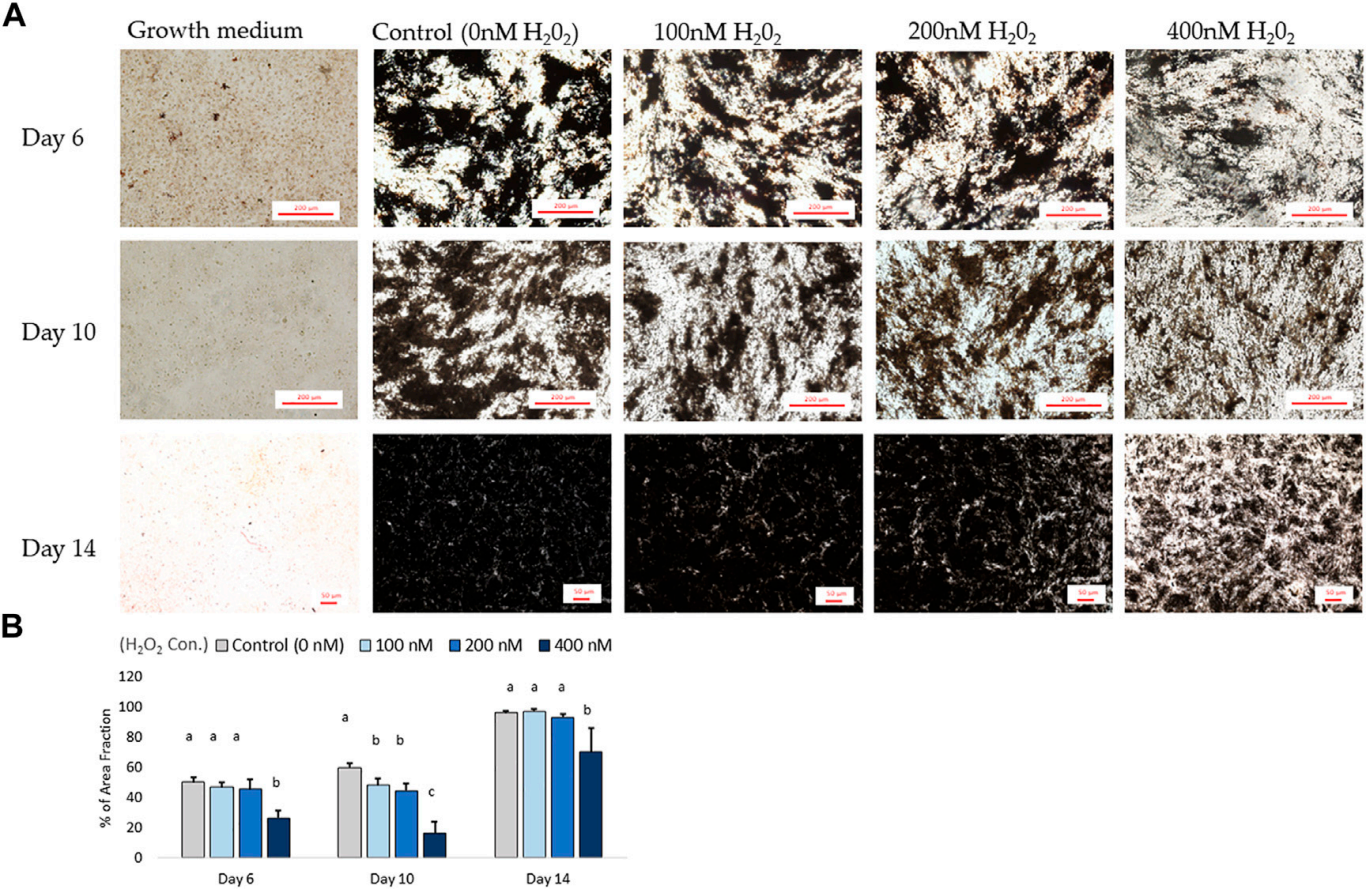
The von Kossa staining results for mineralization on day 6, day 10 and day 14. **(A)** Images were randomly acquired in ×4 magnification for day 6 and 10 images. On day 14, the figure was acquired at ×2 magnification due to mineralization interrupting autofocus using higher magnification lenses. Four images per well were analyzed. Black objects indicates phosphate and calcium deposition. ImageJ analysis quantified the percent area fraction for each treatment based on three independently sampled experiments of each species, with each value representing the mean ± SEM of three independent experiments (*n* = 3). ^a, b^ Treatments with different letters indicate a significantly difference between treatments using Tukey’s HSD test at each time points, respectively; *p* < 0.05.

In parallel with the mRNA expression mentioned above, the inhibition of osteogenic differentiation was characterized by a reduction in mineral accumulation after 6 days, 10 days and 14 days of differentiation. The effects of H_2_O_2_ on the mineralization were visualized using Alizarin red staining (Figure 6A). The optical density (O.D.) value result showed that 400 nM of H_2_O_2_ significantly reduced the O.D. value by 30% compared to the control after 6 days of H_2_O_2_ exposure (*p* < 0.05; Figure 6B). There were no statistically significant changes after 10 and 14 days of differentiation, but smaller mineralized crystals were observed with a higher dose of H_2_O_2_ treatment, and 400 nM H_2_O_2_ led a decrease in mineralization by 20% and 40%. The extracellular calcium (black crystals) content was quantified by von Kossa staining (Figure 7). After 6 days of differentiation, there were smaller and fewer extensive crystals and less mineralized matrix with higher doses of H_2_O_2_ treatment. The colorimetric analysis showed a significant decrease in O.D. with 400 nM H_2_O_2_ treatment (*p* < 0.05; Figure 7B). After 10 days of H_2_O_2_ treatment, a lower number of mineralized nodules and smaller size of crystals were observed with higher concentrations of H_2_O_2_ treatment; the O.D. showed that 200 and 400 nM H_2_O_2_ led to a significant decrease in mineralization, with the least mineral deposition observed in the 400 nM H_2_O_2_ group (*p* < 0.05; Figure 7B). After 14 days of differentiation, 400 nM H_2_O_2_ significantly suppressed mineralization compared to the other groups (*p* < 0.05; Figure 7B). In conclusion, the different doses of H_2_O_2_ treatment resulted in varying effects on osteogenic gene expression at different time points, while mineralization was significantly reduced by H_2_O_2_ treatment, with the greatest reduction observed in the high treatment concentration groups after 10 and 14 days of differentiation.

Meanwhile, under the condition of osteogenic induction differentiation, the expression of adipogenic-related genes was altered with H_2_O_2_ treatment. Decreases in *PPARG* expression with 200 nM and 400 nM H_2_O_2_ treatment doses were observed at 6 h (*p* < 0.05; Supplementary Figure). Afterward, H_2_O_2_ reduced the expression of *PPARG* (*p* = 0.053; Supplementary Figure)*, CEBPA* (*p* < 0.05) and *FABP4* (*p* < 0.05) after 24 h of osteogenic differentiation. In contrast, with prolonged treatment periods, 400 nM H_2_O_2_ significantly elevated the expression of *PPARG* (*p* < 0.05) and *FABP4* (*p* < 0.05) after 96 h of treatment. A similar expression pattern was observed after 5 days of H_2_O_2_ treatment under osteogenic differentiation condition, where 400 nM H_2_O_2_ significantly increased the expression of *PPARG* (*p* < 0.05), *CEBPA* (*p* < 0.05) compared to the control; the expression level of *FABP4* was significantly elevated by 200nM and 400 nM H_2_O_2_ treatment (*p* < 0.05) compared to the control. After 6 days of H_2_O_2_ treatment, 200 and 400 nM H_2_O_2_ treatments increased the expression of *PPARG* (*p* < 0.05; Supplementary Figure) compared to the control; 400 nM H_2_O_2_ treatment drastically increased the expression of *FABP4* (*p* < 0.05; Supplementary Figure) compared to other groups. However, 100 nM H_2_O_2_ reduced expression of *CEBPA* (*p* < 0.05; Supplementary Figure) compared to the control. Adipogenic expression was not significantly changed by H_2_O_2_ treatment on day 10 and day 14.

## Discussion

Cell fate with the presence of oxidative stress can vary depending on the cell types, treatment intensity, duration, dosage, and the cell differentiation status (Denu and Hematti, 2016). Moreover, several studies have pointed out that undifferentiated stem cells have superior antioxidant defense than differentiated cells; for example, undifferentiated MSCs are known to have relatively low levels of intracellular ROS and high levels of glutathione in the human cell line (Valle-Prieto and Conget, 2010). Studies have shown that mouse embryonic stem cells exhibit high antioxidant activity and stress-resistance, but several antioxidant and cellular resistance genes are downregulated during differentiation (Saretzki et al., 2004). Under normal circumstances during mineralization, MSCs differentiate and their expression of mineralization factors increases (Blair et al., 2017). The size of mineral crystals increases during bone mineralization, and the collagen fibers become more organized and condensed (Blair et al., 2017). Studies on other cell types have indicated that H_2_O_2_ solutions have a significant effect on collagen production (Nashchekina et al., 2021), as previous studies have reported the increased activities of oxidative stress was linked to decreased collagen synthesis in fibroblasts (Siwik et al., 2001), human cartilage (Altindag et al., 2007), and chick embryo tissue culture (Ramp et al., 1987). Moreover, the chicken embryo tissue culture has also indicated that multiple exposures to H_2_O_2_ markedly inhibit collagen synthesis (Ramp et al., 1987). In this report, exogenous H_2_O_2_ (100–400 nM) suppressed the osteoblastic mineralization of chicken compact bone-derived MSCs, manifested by reduced osteogenic differentiation gene markers and less mineral deposition. The decreased expression of *Col1A2* after 6 days of H_2_O_2_ treatment supported the hypothesis that H_2_O_2_-induced oxidative stress can directly interrupt type 1 collagen production. Moreover, the concentration of H_2_O_2_ in the present study is much lower than the H_2_O_2_ doses used in human and mouse stem cell studies (Nouri et al., 2019), demonstrating that chicken compact bone-derived MSCs are relatively sensitive to oxidative stress compared to other cell types in chicken (chicken cardiomyocytes: 0.2 mM H_2_O_2_ (Jiang et al., 2005; Wan et al., 2016); chicken cardiac cells, 0.2–2.0 mM H_2_O_2_; and chicken epithelial cells, 300 μM H_2_O_2_ (Lin et al., 2016)). Interestingly, a previous study indicated that ROS production did not influence the aging process of avian fibroblast cells (Strecker et al., 2010). Comparing the blood redox state markers of 78 free-living avian species revealed that relatively long-lived bird species had high levels of antioxidants status (especially total antioxidant status and total glutathione) and low levels of ROS (Xia and Møller, 2018). With the rapid growth and relatively short lifespan of broilers, it is likely that high levels of ROS due to oxidative stress may occur in a great extent in broiler production. Chicken MSCs-differentiated osteoblasts are particularly susceptible to oxidative stress, making the negative impact of ROS production on bone homeostasis a potential factor in the development of skeletal abnormalities.

MSCs have the ability to differentiate into various cell phenotype types, which are controlled by transcription factors such as *PPARG*, *RUNX2* and *SOX9*, which regulate adipogenesis, osteogenesis and chondrogenesis, respectively (Robert et al., 2020). In particular, adipogenesis and osteogenesis have a reciprocal relationship (Takada et al., 2007; Robert et al., 2020). For example, in human and mouse primary MSCs, *PPARG2* insufficiency resulted in increased osteogenesis of osteoblast (Takada et al., 2007), while depletion of *RUNX2* promoted adipogenesis (Enomoto et al., 2004). ROS level in MSCs also plays a crucial role in determining their differentiation potential (Ho et al., 2013; Denu and Hematti, 2016). Previous studies have shown that mRNA expression of antioxidant enzymes such as *SOD, CAT,* and *GPX* is upregulated during adipogenesis in human MSCs (Ho et al., 2013). In this study, prolonged exposure to H_2_O_2_ increased cellular oxidative stress and increased the basal expression of adipogenic differentiation markers at the later stages of differentiation, which was accompanied by decreased mineralization. This is consistent with previous studies that have shown that H_2_O_2_ exposure altered the differentiation potential in human and mouse MSCs or cell lines (Ho et al., 2013; Lin et al., 2018). In addition, by analyzing genome-wide gene expression profiling, Menssen et al. (Menssen et al., 2011) reported an upregulated *CASP8* level during adipogenic differentiation in human bone marrow-derived MSCs. Therefore, the increased expression of *CASP8* on day 6 might have been due to adipogenic differentiation of chicken MSCs, rather than apoptosis directly caused by H_2_O_2_-induced oxidative stress. Moreover, the effect of H_2_O_2_ treatment on regulating cell differentiation has been observed in different types of cells, where sublethal doses of oxidative stress induce morphological alterations (Shadel and Horvath, 2015; Diebold and Chandel, 2016; Infante and Rodriguez, 2018). For example, prolonged H_2_O_2_ treatment activates NF-κB transcriptional activity while stimulating brown adipogenesis during myogenic differentiation in mice satellite cells (Morozzi et al., 2017). Therefore, at least in part, oxidative stress is a factor for the dysfunction of bone tissue, not only by causing cell death, but also by interrupting MSCs differentiation capacity and decreasing the osteogenic ability directly.

In the current study, several osteogenic differentiation markers were significantly upregulated with the highest H_2_O_2_ treatment dose after 4 and 5 days of treatment, and then drastically dropped after 6 days of treatment. We made several hypotheses to explain the upregulated and downregulated expression patterns. Firstly, studies pointed out that the cellular effects of ROS may differ depending on the cell differentiation stage due to the difference between progenitor cells and mature cells (Khalid et al., 2020). For example, during the initial differentiation process, MSCs commit to pre-osteoblasts while actively proliferating (Infante and Rodriguez, 2018). Over the later stage of differentiation, the pre-osteoblasts can further mature into non-proliferating osteoblasts that start matrix secretion, maturation, and mineralization (Infante and Rodriguez, 2018). Studies showed that H_2_O_2_ treatment significantly enhance bone marrow MSCs proliferation and migration ability (Pendergrass et al., 2013). Human and mouse studies revealed a low level of intracellular ROS and high levels of antioxidants in undifferentiated MSCs (Hu et al., 2018). In contrast, differentiated MSCs show a higher level of ROS and lower activity of antioxidative enzymes (Hu et al., 2018). Therefore, it is important to distinguish the multiple roles of ROS in pre-osteoblast differentiation and osteoblast maturation. Secondly, we hypothesize that ROS over-production mediated the cell cycle and caused cell prematurity. ROS is a fundamental signal in many signaling pathways metabolisms (Shadel and Horvath, 2015). The low level of ROS allowed reversible oxidative modifications until the ROS production overwhelm its antioxidant capacity, which leads to severe cellular damage (Diebold and Chandel, 2016). Redox status plays a vital role in the cell cycle, and accumulated intracellular ROS can force MSCs to undergo cellular senescence, substantially interrupt stem cells differentiation (Brandl et al., 2011). The expression of *GPX*, *CAT* and *SOD2* changed in current study, indicating an altered oxidation-reduction status. *SOD2*, which plays a vital role in regulating mitochondrial stress and osteoblastogenesis, was upregulated (Gao et al., 2018). This helped reduce mRNA over-expression of *PPARG and FABP-4* in diabetic mouse models (Sen et al., 2015). In the current study, the increased expression of *SOD2* suggested that cells were actively suppressing adipogenic differentiation under oxidative stress. However, we speculated that long-term exposure cells to higher levels of exogenous H_2_O_2_ stimulated intracellular ROS production and promoted pre-osteoblast commitment at the early differentiation stage, but also led to the accumulation of ROS, which resulted in cell prematurity and apoptosis, decreasing mineral accrual at the later stage. The molecular mechanisms by which ROS affects the avian cell cycle, however, are largely unexplored and require further investigation.

Another hypothesis is that exogenous H_2_O_2_ stimulated a regeneration response. H_2_O_2_ is well-known ROS signaling intermediate in response to tissue injury (Sanchez-de-Diego et al., 2019). ROS activates signaling that influences vital pathways, such as Wnt or TGF/BMP pathways, which are response to fracture healing and tissue repairment mechanism by regulating osteogenic differentiation of stem cells (Wang et al., 2017; Sheppard et al., 2022). MSCs can migrate to the sites of injury in response to various stimuli, including cytokines or growth factors, and differentiate into tissue-specific cell types to repair the damaged region (Merimi et al., 2021). Additionally, ROS can regulate the activation of BMPs and RUNX2 pathways in MSCs during the repair process by mediating the activity of NF-κB signaling (Mandal et al., 2011). Therefore, in the present study, the increased expression of bone formation marks *ALP*, *SPP1* and *BGLAP* after 5 days of differentiation may suggested that the high ROS level stimulated a cell repair-response, which recruited MSCs to differentiate into osteoblasts to maintain cell population and homeostasis. However, continuous oxidative stress cause MSCs to commit apoptosis, ultimately reducing mineralization.

Oxidative stress has been linked to many bone-related diseases in humans and mammals (Reis and Ramos, 2021). In broilers, at least 30% of birds showed poor locomotion during the fast growth period (Wideman, 2016; Zhang et al., 2020), which interfered chicken’ accessibility to feed and water, predominantly reducing the growth and causing an economic loss in production. Generally, cultured cells have higher throughput and shorter turnaround times than *in vivo* study models (Dawson et al., 2014), and the response of avian stem cells to different stress stimuli has been widely studied in the context of growth and physiology (Ali Hassan and Li, 2021; Xu et al., 2022). Therefore, understanding oxidative stress in a cell model is essential for a better understanding of bone pathogenic process in chickens. Tibial dyschondroplasia (TD) and bacterial osteomyelitis (BCO) are two common skeletal abnormalities in the broiler production that cause bones deformation and lameness (Hartcher and Lum, 2019). Previous studies have indicated that BCO is initiated by mechanical micro-fracturing, followed by bacterial colonization and bone degradation, leading to necrosis (Wideman, 2016). Mitochondrial dysfunction and apoptosis are also involved in BCO in broilers (Ferver et al., 2021). Although there is no direct evidence linking oxidative stress and BCO pathogenesis, higher levels of oxidative stress in response to local infection have been reported in human patients with chronic osteomyelitis (Massaccesi et al., 2022). The pathology of osteomyelitis is characterized by localized inflammation, bone mineral loss, and structural damage, which share similarities with broiler BCO (Grbic et al., 2014). Furthermore, a recent study on femoral necrosis pathogenicity also reported an abnormal increased in lipid metabolism and decreases in bone formation in a chicken femoral head necrosis disease model (Fan et al., 2021). Therefore, by gathering all the evidence above, we proposed that oxidative stress could potentially be a co-factor involved in chicken bone necrosis. Moreover, tibial dyschondroplasia (TD) is another common bone abnormality in fast-growing broilers, characterized by tibial bone deformities with non-vascular, non-mineralized growth plates (Mehmood et al., 2017; Zhang et al., 2019). It is a chondrogenesis-related growth plate development disease that is highly associated with premature and apoptosis of cells (Mehmood, 2018). There is an known relationship between TD and oxidative stress induced by thiram, which reduces liver antioxidation capability and damages liver function (Li et al., 2007). Altered systemic antioxidant activity has also been reported in broilers with TD (Zhang et al., 2018; Huang et al., 2021). Although the function of osteoblast has not been fully illustrated in broiler TD model, osteoblasts are responsible for forming the type I collagen matrix surrounding vasculature buds, and osteoblast and osteocyte have direct association with chondrocyte maturation and hypotrophy (Zhang et al., 2020). Based on this information, we hypothesize that TD may be partially associated with systemic oxidative stress. Studies have shown that *mycoplasma* (M.) can produce H_2_O_2_ and superoxide radicals, which induce oxidative stress in the respiratory epithelium and directly affect bone metabolism (Shimizu, 2016). Clinical signs of *M. synoviae* infection include joint lesions in avian species (Osorio et al., 2007). These results provide evidence of the pathogenicity of mycoplasmas on bone integrity and support our current results showing that high level and long-term effects of H_2_O_2_ negatively regulate osteoblast cell activity.

Although many questions remain, ever-growing numbers of observations regarding chicken bone disorders and avian bone health rapidly shape our understanding of various topics, such as metabolic regulation and the pathogenesis of bone disorders in broilers (Porto et al., 2015). The knowledge of ROS generation and antioxidant defense systems has generated a great deal of interest due to its potential applications in animal production, but it remains to be profoundly explored in chicken stem cell models. In conclusion, the concentration of ROS is in a dynamic equilibrium and is modulated by cellular processes that produce and eliminate ROS. Cellular effects of ROS may vary depending on the differentiation stage of the cells. Treatment with H_2_O_2_ altered the expression of cellular antioxidant enzyme gene, and long-term treatment with H_2_O_2_ inhibited osteogenic biomineralization and decreased the expression of osteogenic differentiation markers in chicken MSCs. The impaired osteogenic differentiation potential was associated with an increased potential for adipogenesis in chicken MSCs under oxidative stress, highlighting that cellular oxidative stress caused by exogenous H_2_O_2_ accumulation modulates stem cell differentiation capacity.

## Data Availability

The raw data supporting the conclusions of this article will be made available by the authors, without undue reservation.
