# Supplementary figures and images for: Impact of exogenous hydrogen peroxide on osteogenic differentiation of broiler chicken compact bones derived mesenchymal stem cells
Source: Front Physiol. 2023 Jan 26;14:1124355. doi: 10.3389/fphys.2023.1124355 (PMC9909420; doi:10.3389/fphys.2023.1124355)

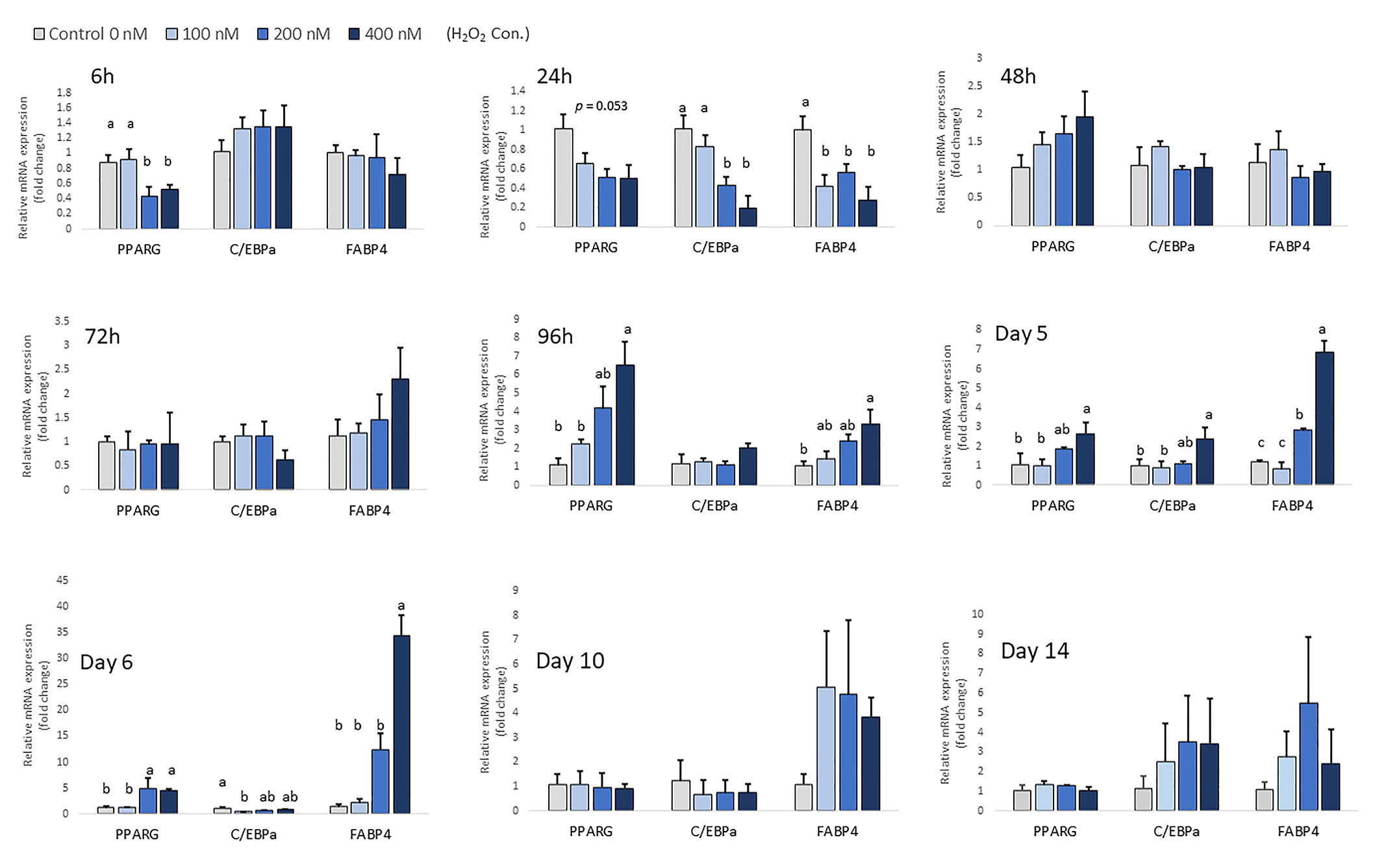

Supplement: Supplementary file 1 [file Image1.TIF]
